# A pre-COVID-19 assessment of aspects of the school health programme in some selected Nigerian primary schools: implications for school re-opening during the COVID-19 pandemic in developing country contexts

**DOI:** 10.1186/s12889-021-11258-x

**Published:** 2021-06-24

**Authors:** Usman A. Sanni, Uduak M. Offiong, Emmanuel A. Anigilaje, Kareem I. Airede, Abdulazeez Imam

**Affiliations:** 1 Department of Paediatrics, Federal Medical Centre, Birnin-Kebbi, Kebbi Nigeria; 2 Department of Paediatrics, University of Abuja Teaching Hospital, Gwagwalada, Abuja Nigeria; 3 Department of Paediatrics, University of Abuja, Gwagwalada, P.M.B 117, Abuja, Nigeria; 4 Vaccines and Immunity Theme, Medical Research Council Unit The Gambia at the London School of Hygiene and Tropical Medicine, Atlantic Boulevard, P.O. Box 273, Fajara, Gambia

**Keywords:** COVID-19, School health program, Pandemic, Nigeria, Developing countries

## Abstract

**Background:**

Following the COVID-19 pandemic, school closures were part of the global public health response to limit community spread of the virus. In recent times, there has been an emphasis on safe school re-opening. This concept is likely to differ between developed and developing country settings. There are however no published studies on barriers hindering safe school re-opening within developing country contexts. This study evaluates aspects of the school health program (SHP) in some selected Nigerian schools that might relate to the pandemic control during school re-opening.

**Methods:**

In 2017, we conducted a cross-sectional survey of the SHP of 146 registered primary schools in Gwagwalada Area Council in Abuja, Nigeria. These schools provided services to about 54,562 students. We used direct observational methods and interviewer-administered questionnaires to assess the SHP of each school. We compare SHP characteristics that might relate to COVID-19 control in schools across government-owned (public) and privately-owned (private) schools using a pre-defined framework.

**Results:**

Public school to pupil ratios was more than six times that of private schools. Only 6.9% of all surveyed schools employed qualified health personnel. Although 8 in every 10 schools conducted health talks for communicable disease control, the use of temporary isolation and school-based immunization were low at 1.4 and 2.7% respectively. Pipe-borne water access was present in 4 of 10 schools, with public schools having more limited access than private schools (*p* = 0.009). Similarly, less proportion of public schools had access to soap for handwashing (*p* < 0.001). Adequate classroom ventilation was present in 63% of surveyed schools, with private schools having more limited ventilation (*p* < 0.001).

**Conclusions:**

Overcrowding and infrastructural deficits within developing country contexts represent barriers to safe school re-opening during the COVID-19 pandemic. In these settings, there needs to be tailored and innovative strategies which consider local practical realities when designing the COVID-19 control programs during school re-opening.

## Background

The COVID-19 pandemic has continued to spread across the globe with over 150 million confirmed cases recorded and almost 3.3 million deaths occurring worldwide as of the 9th of May, 2021 [1]. At the onset of the pandemic, countries initiated lockdowns and other public health measures including school closure to curtail the virus spread. This resulted in the closure of schools in more than 165 countries, with consequent interruption of the learning process of almost 1.5 billion children [2]. Most recently, governments are beginning to ease restrictions and schools have now re-opened allowing children to continue their education and also limiting the negative effects that might arise from prolonged school closure. It is thought that extended periods of school closure might impact both the mental and physical health of school children [3, 4]. It might also affect programs such as vaccination, school feeding and mass de-worming which are routinely delivered through the school health program in low-middle-income countries (LMICs) and can potentially reverse the gains on female education in these settings [5].

In line with international best practices, the Nigerian national Ministry of Education officially ordered school closure on the 3rd March 2020 and recently been re-opened on the 12th of October 2020 [6]. This is also a similar situation in many other developing country contexts [7]. Global school re-opening is premised on current scientific evidence which demonstrates that children do not transmit the COVID-19 virus as efficiently as adults do, and that school-based virus transmission may not be the main driver of community transmission [8, 9]. Also, epidemiologic studies report children to have a predominantly asymptomatic or milder illness when compared to adults [10–12] and overall better disease prognosis [12]. With this evidence, medical societies have advocated for the safe re-opening of schools with appropriate precautionary measures such as hand hygiene, reduced intermixing, physical distancing and the use of face masks [13]. The World Health Organization has also released a statement to guide the safe re-opening of schools [14]. Requirements for schools in developing countries to establish safer school environments might however differ from those in more developed countries as the former have a comparatively greater deficit in infrastructure and their school health programs. Locally, in Nigeria, the National Center for Disease Control (NCDC) has brought out a policy that directs schools on appropriate procedures to follow including ensuring adequate classroom ventilation, social distancing within schools and wearing of masks in children above the age of six [15]. In reality, there might exist significant barriers that hinder the adoption of these public health measures.

**Fig. 1:**
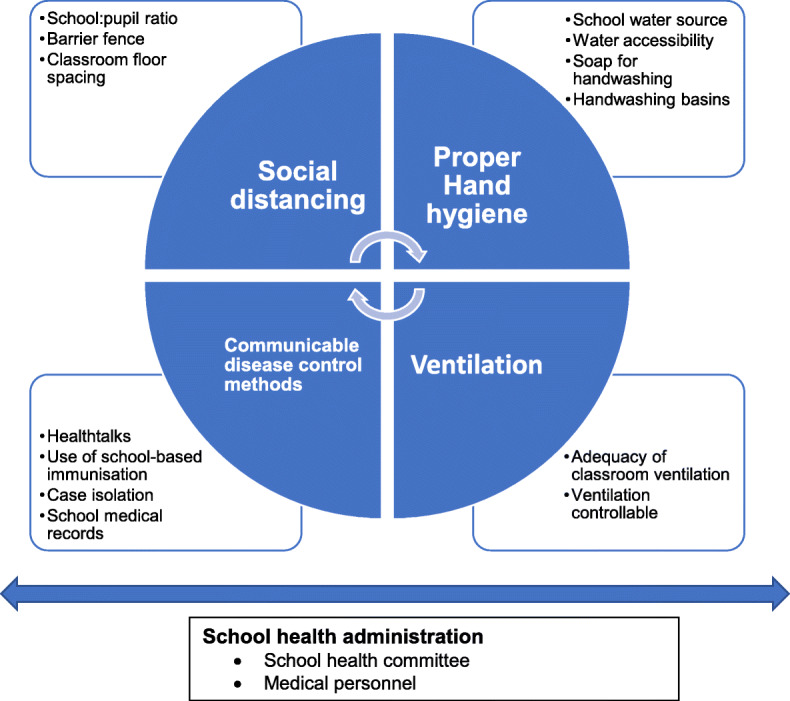
Framework for determining study variables. Four parts of the cycle represent core Covid-19 strategies and the attached squares represent proxy measures for this (The school health administration controls the implementation of strategies)

To the best of our knowledge, there are no published context-specific studies that identify deficits in developing countries’ school health programs (SHPs) that need to be addressed to ensure safe school re-opening during the pandemic. Using previously collected data on the school health program of 146 selected primary schools in the Nigerian capital, we identified gaps in the school health program regarding COVID-19 mitigation preparedness using the framework in Fig. 1. Such data is important to inform practical strategies for mitigating COVID-19 in Nigerian schools and potentially in other developing country contexts. This is also important if these schools are to remain open during the subsequent infection waves as currently being experienced globally.

## Subjects and methods

### Study design and setting

We conducted a cross-sectional survey using methods of direct observation and interviewer-administered questionnaires **(**Table 1**)** between April to October 2017. Our study was conducted in the Gwagwalada Area Council (GAC) of the Federal Capital Territory (FCT), Nigeria. The GAC is one of six area councils in the FCT, located in North-central, Nigeria and has a population of 402,000 [16]. The Universal Basic Education (UBE) Board and Zonal Education Office (ZEO), Gwagwalada are in charge of primary school education within the Area Council. In 2017, the GAC had 291 registered primary schools comprising 80 public and 211 private schools.

**Table 1:** Study variables and the methods of inspection

Variables	Methods of inspection
**Presence of a health personnel**	Direct observation
**Water supply**	Direct observation
**Presence of a school fence**	Direct observation
**Classroom ventilation**	Direct observation
**Availability of soap**	Direct observation
**Availability of wash hand basin**	Direct observation
**Sickbay**	Direct observation
**Floor spacing**	Measurements taken
**Adequacy of ventilation**	Measurements taken
**Controllable ventilation**	Direct observation
**Student medical records**	Direct observation
**Presence of school health committee**	Questionnaire
**Conduct of health talk**	Questionnaire
**The practice of temporarily isolating sick children**	Questionnaire
**Immunization practice**	Questionnaire

### Study population

These were private and public schools in the GAC.

### Sampling technique and sample size determination

To calculate our sample size, we used a sampling ratio of 50% of our sampling frame to arrive at the largest possible sample for a chosen error margin of 0.05 [17]. The sampling frame comprised all 291 registered schools, thus we arrived at a sample size of 146 schools. We used stratified random sampling to select our chosen schools by first stratifying all schools into public and private schools and then selected 50% in each school group (i.e. 40 public and 106 private schools) using simple balloting.

### Data collection

To access the school health program of individual schools, we used an SHP evaluation tool that was modified for use in Nigerian schools by Azubuike et al. [18] This tool comprised sections to evaluate all aspects of the SHP (school healthful environment, school health instruction, school medical services and school health administration) [18]. For the current research, we have selected a sub-set of variables based on our framework in Fig. 1. These were determined using a combination of methods of direct observation, questionnaire administration and also taking measurements (Table 1). The research team comprised a paediatrician and four final year undergraduate students. To ensure data consistency and quality, the team was trained by the paediatrician for 4 weeks. In each school, we administered our questionnaires to the headteachers and a random selection of three school teachers. We further selected two sixth form pupils (the highest form in the Nigerian primary school) to confirm findings from the questionnaires administered to the teachers (Table 1). Also, we carried out direct observation/inspection of other aspects of the SHP and measured windows, floor spaces and doors in classrooms using an inelastic measuring tape (Table 1).

### Exposure variable definitions

1. Adequate ventilation

This was defined as doors and windows with a combined area that accounted for at least 25% of the floor space and allowed for cross ventilation by windows being placed on different classroom walls [19].

2. Controllable ventilation

Ventilation was adjudged controllable where doors and windows had hinges that allow for easy opening and closing.

3. School health committee

The committee comprising of headteacher (as chairman), pupils’ representative, school nurse, health teacher, and representative of Parent-Teachers Association. The committee is to ensure the implementation of the SHP at the schools [20].

4. Standard classroom floor spacing

Floor classroom spacing was considered standard if it was at least 19.4 m^2^ and accommodated a maximum of thirty-six pupils arranged in six rows and six columns [21]. This was definition was based on a policy guideline document for school sanitation by the Nigerian Federal Ministry of Environment [21].

### Outcome variable

School Type – Public or private school

1. Public schools were defined as schools established and owned by the government.
2. Private schools were defined as schools established and owned by an individual, or group of persons, organizations or mission bodies.

### Ethical considerations

Ethical approval for our study was obtained from the Research and Ethics Committee of the University of Abuja Teaching Hospital’s (FCT/UATH/HREC/PR/034). We also received study approvals from the FCT Universal Basic Education Board, Zonal Education Office and the GAC. We also sought consent from all study participants and caregivers of participating students. Additionally, we anonymised data collection through the use of codes on the school assessment forms.

### Data analysis

We determined the school to pupil ratios by dividing the number of schools by pupils across both public and private schools. We also determined frequency and proportions for each variable across the two groups. We compared proportions using chi-square and Fischer’s exact when an expected cell count was less than 5. Statistical significance was set at *p* < 0.05 for our univariable analysis. All statistical analyses were performed using STATA version 16.1 (StataCorp. 2019. Stata Statistical Software: Release 16. College Station, TX: StataCorp LP).

## Results

We conducted our study in 146 (50.2%) of the 291 registered primary schools in the GAC. Of this number, 106 (72.6%) were privately owned, while 40 (27.4%) were government-owned, referred here on out as ‘private’ and ‘public schools’ respectively. These schools provided services to a combined school population of 54,562 students comprising 26,849 males and 27,708 females. Private schools’ students had a student population of 15, 881, while public schools had a population of 38, 681. Overall average pupil to school ratio was 373.7: 1. Public primary schools had an average pupil to school ratio of 967:1, while private primary schools had a ratio of 149.8: 1.

### School health processes compared between public and private schools

About 21.2% of surveyed schools had a school health committee with significantly more public health schools (40.0%) having such committees when compared to private schools (14.2%, *p* = 0.001, Table 2). Similarly, only 6.9% of all the surveyed schools employed medical personnel with no public school having employed medical personnel (Table 2).

**Table 2:** School health processes compared between public and private schools

Variable	Public schools (*n* = 40)	Private school (*n* = 106)	*p*-value	Total
**Availability of a school health committee**				
Yes	16 (40.0)	15 (14.2)	**0.001**	31 (21.2)
No	24 (60.0)	91 (85.9)		115 (78.8)
**Availability of health personnel**^**a**^				
Yes	0 (0.0)	10 (9.4)	0.06	10 (6.9)
No	40 (100.0)	96 (90.6)		136 (93.2)
**Conduct health talks**				
Yes	34 (85.0)	88 (83.0)	0.77	122 (83.6)
No	6 (15.0)	18 (17.0)		24 (16.4)
**Isolate school-children as a method for disease control**				
Yes	1 (2.5)	1 (0.9)	0.47	2 (1.4)
No	39 (97.5)	105 (99.1)		144 (98.6)
**Immunise school- children as a method for disease control**				
Yes	2 (5.0)	2 (1.9)	0.46	4 (2.7)
No	38 (95.0)	104 (98.1)		142 (97.3)
**Keep student medical records**				
Yes	23 (57.5)	68 (64.2)	0.46	91 (62.3)
No	17 (42.5)	38 (35.9)		55 (37.7)

^**a**^Doctor or nurse

For the control of communicable diseases within the school environment, the majority (83.6%) of schools conducted health talks (Table 2). Fewer schools carried out temporary isolation at school (1.4%) or conducted school-based immunisation services (2.7%) (Table 2).

### School physical environment compared between public and private schools

Among surveyed schools, 39.7% of schools had access to pipe-borne water. This access was significantly higher in private schools (46.2%) when compared to public schools (22.5%, *p*-value = 0.009, Table 3). A greater proportion of public schools, however, had a bore-hole as the main source of water when compared to private schools (*p* = 0.02, Table 3). Also, a greater proportion of government schools (70.0%) had their water sources within the school premises when compared to those of private schools (48.1%, *p*-value =0.018). As regards classroom ventilation, government schools on average had better ventilation when compared to private schools (Table 3). Overall, 75.3 and 52.7% of schools had access to soap for handwashing and school wash hand facilities respectively. These proportions were significantly lower in public schools when compared to private schools (Table 3, *p* = < 0.001). No surveyed public school had a health room or sickbay, while 23.6% of private schools had one (Table 3).

**Table 3:** School physical environment compared between public and private schools

Variable	Public schools (*n* = 40)	Private school (*n* = 106)	*p*-value	Total
**Access to pipe-borne water**				
Yes	9 (22.5)	49 (46.2)	**0.009**	58 (39.7)
No	31 (77.5)	57 (53.8)		88 (60.3)
**Borehole as the main water source**				
Yes	27 (67.5)	48 (45.3)	**0.03**	75 (51.4)
No	13 (32.5)	58 (54.7)		71 (48.6)
**Well water as the main water source**				
Yes	1 (2.5)	10 (9.4)	0.16	11 (7.5)
No	39 (97.5)	96 (90.6)		135 (92.5)
**Surface water as the main water source**				
Yes	3 (7.5)	1 (0.9)	**0.03**	4 (2.7)
No	37 (92.5)	105 (99.1)		142 (97)
**School fenced**				
Yes	12 (30.0)	91 (85.9)	**< 0.001**	103 (70.6)
No	28 (70.0)	15 (14.2)		43 (29.5)
**School water source location**				
Within school premises	28 (70.0)	51 (48.1)	**0.018**	79 (54.1)
Outside school premises	12 (30.0)	55 (51.9)		67 (45.9)
**Ventilation in classrooms**				
Adequate	38 (95.0)	54 (50.9)	**< 0.001**	92 (63.0)
Not adequate	2 (5.0)	52 (49.1)		54 (37.0)
**Ventilation source in classrooms**				
Controllable	37 (92.5)	92 (86.8)	0.34	129 (88.4)
Not controllable	3 (7.5)	14 (13.2)		17 (11.6)
**Classroom floor spacing**				
Standard	40 (100.0)	34 (32.1)	**< 0.001**	74 (50.7)
Non-standard	0 (0.0)	72 (67.9)		72 (49.3)
**Presence of wash hand basins within schools**				
Yes	2 (5.0)	75 (70.8)	**< 0.001**	77 (52.7)
No	38 (95.0)	31 (29.3)		69 (47.3)
**Availability of soap for handwashing**				
Yes	8 (20.0)	102 (96.2)	**< 0.001**	110 (75.3)
No	32 (80.0)	4 (3.8)		36 (24.7)
**Presence of sickbay/ health room**				
Yes	0 (0.0)	25 (23.6)	**0.001**	25 (17.1)
No	40 (100.0)	81 (76.4)		121 (82.9)

Bold – statistically significant

## Discussion

Using data from a previous school health program (SHP) survey, this paper identifies potential barriers to COVID-19 mitigation preparedness in some selected Nigerian primary schools and compares these barriers across public and private schools. Identified barriers that affected core COVID-19 prevention strategies such as performance of hand hygiene in schools, classroom ventilation, social distancing, and the control of communicable diseases.

Proper hand hygiene is central to COVID-19 prevention and involves frequent hand-washing with soap or the use of alcohol-based hand rubs as a substitute when water is unavailable [22]. In the surveyed schools, we found structures that might promote handwashing were limited and this limitation was greater in public schools. Overall, only 4 out of 10 schools had pipe-borne water with public schools having less access (2 in 10 schools). Fewer public schools also had handwashing facilities within their premises. In contrast to other factors that might enhance hand hygiene, water source accessibility was greater in public schools, as more of these schools had their water sources within the school premises. These were predominantly boreholes built by the government under a previous Millennium Development Goals (MDG) project. Our findings of limited handwashing access in schools are similar to those from studies conducted in other parts of Nigeria and other similar settings to ours [23–25]. A survey by UNICEF found only 46% of schools in developing countries had access to adequate water sources [25]. A previous Kenyan study contrasted our findings by demonstrating 60% of surveyed schools have adequate handwashing facilities, but noted high support by Non-governmental organisation for Water, Sanitation and Hygiene (WASH) practices in these schools [26]. In developing countries, the availability and accessibility of water and soap for handwashing within schools are strongly associated with good school handwashing practices [27–29]. For successful pandemic control during school re-opening, greater focus needs to be placed on improving soap and water availability, particularly within public schools, where our data shows the greater deficit. Existing infrastructural deficits might be substantial to reverse in the short term, and might require a phased intervention. In the interim however, alcohol-based hand sanitisers can be provided in classrooms, while heavy investments in school water provision is carried out in conjunction with development partners and this should ideally extend to supporting private schools. Adequate water provision in schools would have important implications outside mitigating COVID-19, such as a reduction in diarrheal associated illnesses, respiratory illnesses, helminthic infections and decreased school absenteeism for adolescent girls through improvements in menstrual hygiene [30, 31].

Our study also found that classroom ventilation was only adequate in less than two-thirds of the surveyed schools with a higher proportion of public schools having adequate ventilation when compared to private schools. This difference most probably relates to differences in the initial purposes for which these schools were built. Locally, public schools which are run by the government are originally built for educational purposes, while it is not uncommon for private schools to rent and operate from structures that were originally residential. For proper COVID-19 control during school re-opening, school classrooms in facilities with inadequate ventilation might need to budget to remodel their doors and windows. To ensure compliance, there needs to be standards and laws put in place by regulatory authorities, particularly as regards adapting structures for schools that were not originally built for such purposes.

In this study, 7 out of 10 public schools had no form of barrier fencing which is crucial for controlling traffic in and out of schools. Barrier fences are central to upholding physical distancing and avoiding overcrowding in schools as they allow for well-defined entry and exit points where movement in and out of schools can be controlled. These areas might also serve as triage points where pupils are screened for high temperature, compliance with wearing masks and performing hand hygiene. To limit overcrowding and promote social distancing within classrooms, the Nigeria Centre for Disease Control (NCDC) recommends that classroom pupils sit one metre apart in line with international regulations [15]. In this study, we employed two indices to determine classroom overcrowding including pupil-school ratios and the adequacy of classroom floor sizes. In this setting, public primary pupil to school ratios was six times those of private primary schools**.** In contrast, less proportion of private schools had standard floor sizes, suggesting some overcrowding within these settings which might also relate to some of these schools being originally built for residential purposes. Other researchers have also described school overcrowding in other developing country settings such as India [32]. Locally, it would be impractical to limit public school intake as a large group of children who can not afford private schooling would be without access to education. While there need to be infrastructural developments in the education sector, the urgency of the situation requires innovative methods that limit overcrowding and ensure some social distancing is maintained within classrooms. The Nigerian Federal Ministry of Education has recognised this challenge and suggested alternate methods of learning, for example, considering outdoor learning, dividing schools into morning and afternoon shifts or different groups of students attending schools on alternate days of the week [33].

Another important aspect for COVID-19 control during school re-opening would be communicable disease control within schools. We assessed three channels for such control including school-based immunisation services, use of health talks and isolation of children with communicable diseases. We found immunisations services, both as a booster for routine vaccination and the control of epidemics were largely non-existent in both surveyed private and public schools, and this is similar to findings from other Nigerian studies [34, 35]. While this finding does not have an immediate implication for COVID-19 control, it potentially impacts future vaccine delivery within schools as an important public health measure to curb the pandemic spread. Current studies might thus need to understand factors that might promote COVID-19 vaccine uptake among school children in developing country contexts, similar to studies previously carried out among health workers and adult populations [36–38]. The current deficit in immunisation services also represents missed opportunities to deliver other existing vaccines, for example, the human papillomavirus vaccine to adolescent females, as is routinely carried out in developed country settings. Additionally, isolation of school children suspected of having a communicable disease was not routinely practised in schools and only 17.1% of schools had a sick bay or health-room within their school with no surveyed public school having one. In a previous study from South-Western Nigeria, a quarter of surveyed schools had a sickbay with surveyed public schools having more limited access [39]. Similarly, another study from Pakistan found only 24% of surveyed schools to have a sickbay [40]. As schools reopen during the pandemic, administrators will need to identify rooms for isolating suspected cases of COVID-19. Importantly, our study identified the majority of schools currently use health talks to deliver messages for communicable disease control to students. This is important, as schools could easily adapt such messages to educate school children about COVID-19.

For successful COVID-19 mitigation within schools, having appropriate human resource and the necessary school health administration is also critical to direct and implement local control strategies. This requires the presence of a school health committee which usually acts as an implementation driver for the SHP and can easily be adapted to provide oversight functions for control of COVID-19. Such committees have been shown to increase the likelihood of attaining minimum school health service deliverables in similar settings to ours [41]. We found that just over a fifth of schools had such committees and the deficit was greater among private schools. Also, schools would need medically qualified personnel to serve as advisers to these committees, play prominent roles in the frontline COVID-19 response through co-ordinating surveillance and promoting a biopsychosocial approach to disease management where the mental health of returning pupils is considered paramount. A recent Chinese study demonstrated anxiety and depression to be around 6 and 12% respectively among children and adolescents returning to schools after lockdown [42]. There is increasing recognition that existing mental health systems would not be able to cope with the fall-out of the pandemic and there are calls for targeted school-based mental health interventions [43]. One area that has shown promise in improving mental health among adults returning to work after lockdowns are psychoneuroimmunity preventive measures [44]. This involves the promotion of COVID-19 safe workspaces and is associated with reduced psychological problems in returning workers [44]. The role of such preventative measures in improving mental health among school pupils in developing countries would need to be the focus of future research. The number of health personnel employed by schools in the study area was markedly limited, as only 6.9% of schools employed either a nurse or doctor and none of the surveyed public schools employed one. This is similar to two Nigerian studies that showed 4.3 and 9.3% of schools employed health personnel [45, 46]. Our figures are however much lower than a previous study from south-western Nigeria which found 23.3% of schools to employ a nurse [39]. Although the findings of this study are at variant with ours, they still show sub-optimal human health resources within schools and possibly suggest regional differences in the strength of SHPs. This suggests an existing gap between policy and actual practice, as the Nigerian national school health policy recommends all schools should have school health service personnel including medical doctors or school nurses [20]. Common in our setting is to have non-medically qualified teachers trained to provide first aid to students. As schools re-open, administrators would need to create health committees if not in existence, or if non-functional, empower them to carry out all aspects of the SHP including COVID-19 control. Also, these schools would need to hire medical professionals. In a context with limited resources like ours, this might be impractical in the short-term and some creative thinking would be needed to address the human resource for health deficit within schools. Countries such as Vietnam are considering the use of village health workers for control of COVID-19 [47]. There is a similar cadre in the form of Community Health Extension Workers (CHEWs) in Nigeria, a lower skilled cadre of health workers who complete 2 to 3 years of formal health training including a year of apprenticeship and mentoring [48]. CHEWs have successfully been deployed to strengthen primary health services, particularly among rural Nigerian communities [49], and could potentially be used to strengthen the local SHP. If such structures are funded and sustained, they could lead to a long-lasting gains for the Nigerian SHP.

### Strengths and limitations

To the best of our knowledge, ours is the first study to identify barriers to safe school re-opening in a developing country context and we have described challenges in our local setting that potentially apply to other developing country contexts.

While data for our analysis were collected in 2017, we suspect that there might have been minimal change in the state of schools. This is because Nigeria had been experiencing an economic recession from falling oil prices even before the start of the pandemic. Additionally, we conducted our study in a large number of schools within one of six area councils within the FCT. Nigeria is however quite expansive and there are possibly some geographical variations in the availability of resources for the school health program. Nevertheless, multiple studies from Nigeria and other developing countries describe deficits in the SHP which are similar to our findings [23, 27, 35, 40], suggesting these contexts might experience similar challenges to ours. Our study does not measure actual class sizes to identify classroom overcrowding but we utilised proxies such as pupil to school ratio which can indirectly measure classroom overcrowding.

## Conclusion

There are multiple barriers to safe school re-opening within the contexts studied. This relates majorly to Infrastructural deficits and gaps in policy to practice. While blueprints currently exist for safe school re-opening, they need to that take into cognisance the local realities that might exist within developing country contexts, many of which arise from limited school infrastructure and human resource. Additionally, standards will need to be set to ensure safe school re-opening during the pandemic and existing policies on the school health program need to be enforced. There also needs to be a concerted strengthening of the local SHP to build resilience not only against COVID-19 but also against future epidemics that might occur.

## Data Availability

Data used in this study are available from the corresponding author upon reasonable request.

## Abbreviations

FCT: Federal Capital Territory
GAC: Gwagwalada Area Council
LMIC: Low-middle income countries
MDG: Millennium Development Goals
NCDC: National Center for Disease Control
SHP: School Health Program
UBE: Universal Basic Education
UNICEF: United Nations Children’s Fund
WASH: Water, Sanitation and Hygiene
ZEO: Zonal Educational Office

## References

[1] Weekly epidemiological update on COVID-19 - 11 May 2021. [cited 2021 May 23]. Available from: https://www.who.int/publications/m/item/weekly-epidemiological-update-on-covid-19%2D%2D-11-may-2021

[2] https://plus.google.com/+UNESCO. Education: From disruption to recovery. UNESCO. 2020 [cited 2020 Nov 9]. Available from: https://en.unesco.org/covid19/educationresponse

[3] Liu JJ, Bao Y, Huang X, Shi J, Lu L (2020). Mental health considerations for children quarantined because of COVID-19. Lancet Child Adolesc Health.

[4] Rundle AG, Park Y, Herbstman JB, Kinsey EW, Wang YC (2020). COVID-19–related school closings and risk of weight gain among children. Obesity..

[5] Viner RM, Bonell C, Drake L, Jourdan D, Davies N, Baltag V, et al. Reopening schools during the COVID-19 pandemic: governments must balance the uncertainty and risks of reopening schools against the clear harms associated with prolonged closure. Arch Dis Child. 2021;106(2):111–3. 10.1136/archdischild-2020-319963 PMC7401577 32747375

[6] Nigeria Government Calls for Reopening of Schools After 6-month COVID Lockdown | Voice of America - English. [cited 2020 Nov 18]. Available from: https://www.voanews.com/africa/nigeria-government-calls-reopening-schools-after-6-month-covid-lockdown

[7] After months of closure, Kenya’s schools adjust to sudden reopening. The World from PRX. [cited 2020 Nov 18]. Available from: https://www.pri.org/stories/2020-10-16/after-months-closure-kenyas-schools-adjust-sudden-reopening

[8] Viner RM, Mytton OT, Bonell C, Melendez-Torres GJ, Ward J, Hudson L, et al. Susceptibility to SARS-CoV-2 infection among children and adolescents compared with adults: a systematic review and meta-analysis. JAMA Pediatr. 2021;175(2):143–56. 10.1001/jamapediatrics.2020.4573 PMC7519436 32975552

[9] Lee B, Raszka WV (2020). COVID-19 transmission and children: the child is not to blame. Pediatrics.

[10] Lu X, Zhang L, Du H, Zhang J, Li YY, Qu J (2020). SARS-CoV-2 infection in children. N Engl J Med.

[11] Dong Y, Mo X, Hu Y, Qi X, Jiang F, Jiang Z (2020). Epidemiology of COVID-19 among children in China. Pediatrics.

[12] Götzinger F, Santiago-García B, Noguera-Julián A, Lanaspa M, Lancella L, Carducci FIC (2020). COVID-19 in children and adolescents in Europe: a multinational, multicentre cohort study. Lancet Child Adolesc Health..

[13] Walger P, Heininger U, Knuf M, Exner M, Popp W, Fischbach T (2020). Children and adolescents in the CoVid-19 pandemic: Schools and daycare centers are to be opened again without restrictions. The protection of teachers, educators, carers and parents and the general hygiene rules do not conflict with this. GMS Hyg Infect Control.

[14] Organization WH (2020). Considerations for school-related public health measures in the context of COVID-19.

[15] Nigerian Centre for Disease Control. Public health guidance on safe school re-opening in Nigeria. [cited 2020 Nov 23]. Available from: https://covid19.ncdc.gov.ng/media/files/Public_Health_Guidance_on_Safe_School_Re-opening_in_Nigeria.pdf

[16] Nigeria NPC of. Federal Capital Territory 2016 Population Projection. 2016 [cited 2017 Sep 11]. Available from: https://www.citypopulation.de/php/nigeria-admin.php?adm1id=NGA015

[17] Lwanga SK, Lemeshow S, Organization WH (1991). Sample size determination in health studies: a practical manual.

[18] Azubuike JC, Nkangineme KE (2016). Paediatrics & Child Health In The Tropics.

[19] Park K. Park’s textbook of preventive and social medicine. 23rd ed. India: Bhanot Publishers; 2015.

[20] National School Health Policy.pdf. Google Docs. [cited 2020 Nov 25]. Available from: https://drive.google.com/file/d/0B1DAmtM1BcbMMU5LY0VNbnl1NlU/view?usp=drivesdk&usp=embed_facebook

[21] Federal Ministry of Environment (2005). Policy guidelines on school sanitation.

[22] Advice for the public on COVID-19 – World Health Organization. [cited 2020 Nov 12]. Available from: https://www.who.int/emergencies/diseases/novel-coronavirus-2019/advice-for-public

[23] Nwajiuba CA, Ogunji CV, Uwakwe RC, David EI (2019). Handwashing practices among children in public schools in Imo state, Nigeria. Glob J Health Sci.

[24] Babalobi B (2013). Water, sanitation and hygiene practices among primary-school children in Lagos: a case study of the Makoko slum community. Water Int.

[25] Joint call to action. Raising Clean Hands: Advancing Learning, Health and Participation through WASH in Schools [Internet]; 2010 [cited 2020 Dec 15]. Available from: https://www.unicef.org/media/files/raisingcleanhands_2010.pdf.

[26] Alexander KT, Oduor C, Nyothach E, Laserson KF, Amek N, Eleveld A, Mason L, Rheingans R, Beynon C, Mohammed A, Ombok M, Obor D, Odhiambo F, Quick R, Phillips-Howard P (2014). Water, sanitation and hygiene conditions in Kenyan rural schools: are schools meeting the needs of menstruating girls?. Water..

[27] Besha B, Guche H, Chare D, Amare A, Kassahun A, Kebede E (2016). Assessment of hand washing practice and it’s associated factors among first cycle primary school children in Arba Minch town, Ethiopia, 2015. Epidemiol Sunnyvale.

[28] Saeed S, Ghebrehiwot L, Juni MH (2018). Factors associated with hand washing practices among adolescents Yemeni students in Klang valley, Malaysia. Int J Public Health Clin Sci.

[29] Bulled N, Poppe K, Ramatsisti K, Sitsula L, Winegar G, Gumbo J, Dillingham R, Smith J (2017). Assessing the environmental context of hand washing among school children in Limpopo. South Africa Water Int.

[30] McMichael C (2019). Water, sanitation and hygiene (WASH) in schools in low-income countries: a review of evidence of impact. Int J Environ Res Public Health.

[31] Lau CH, Springston EE, Sohn M-W, Mason I, Gadola E, Damitz M (2012). Hand hygiene instruction decreases illness-related absenteeism in elementary schools: a prospective cohort study. BMC Pediatr.

[32] Majra JP, Gur A (2010). School environment and sanitation in rural India. J Glob Infect Dis.

[33] Federal Ministry of Education. Guidelines for schools and learning facilities reopening after covid-19 pandemic closures. 2020 [cited 2020 Nov 24]. Available from: https://covid19.ncdc.gov.ng/media/files/COVID_19_GUIDELINES_FOR_SAFE_REOPENING.pdf

[34] Olatunya O, Oseni S, Olaleye A, Akani NOO (2015). School health Services in Nigeria : a sleeping giant ?. Afr J Health Sci.

[35] Bisi-onyemaechi AI, Akani AN, Ikefuna AN, Tagbo BN, Chinawa JM, Chikani UN (2017). School health services in Enugu east, Nigeria : perspectives from a resource-poor setting. Healthc Low -Resour Settings.

[36] Chew NW, Cheong C, Kong G, Phua K, Ngiam JN, Tan BY (2021). An Asia-Pacific study on healthcare workers’ perceptions of, and willingness to receive, the COVID-19 vaccination. Int J Infect Dis.

[37] Dodd RH, Cvejic E, Bonner C, Pickles K, McCaffery KJ, Ayre J (2021). Willingness to vaccinate against COVID-19 in Australia. Lancet Infect Dis.

[38] Chen M, Li Y, Chen J, Wen Z, Feng F, Zou H, et al. An online survey of the attitude and willingness of Chinese adults to receive COVID-19 vaccination. Hum Vaccines Immunother. 2021;17(7):2279–88. 10.1080/21645515.2020.1853449 PMC8189089 33522405

[39] Kuponiyi OT, Amoran OE, Kuponiyi OT (2016). School health services and its practice among public and private primary schools in Western Nigeria. BMC Res Notes.

[40] Nasim S (2018). School health services and its practices in public and private schools of Rawalpindi District. J Islamabad Med Dent Coll.

[41] Bowman AS, Owusu A, Trueblood AB, Bosumtwi-Sam C (2018). Impact of school health management committees on health services delivery in Ghana: a national level assessment. Int J Health Plann Manag.

[42] Liu Y, Yue S, Hu X, Zhu J, Wu Z, Wang J, Wu Y (2021). Associations between feelings/behaviors during COVID-19 pandemic lockdown and depression/anxiety after lockdown in a sample of Chinese children and adolescents. J Affect Disord.

[43] Hamoda HM, Chiumento A, Alonge O, Hamdani SU, Saeed K, Wissow L, Rahman A. Addressing the Consequences of the COVID-19 Lockdown for Children’s Mental Health: Investing in School Mental Health Programs. Psychiatr Serv. 2021. Available from: 10.1176/appi.ps.202000597. Accessed 2021 Jun 1. [Epub ahead of print]. 10.1176/appi.ps.202000597 PMC8192330 33502220

[44] Tan W, Hao F, McIntyre RS, Jiang L, Jiang X, Zhang L (2020). Is returning to work during the COVID-19 pandemic stressful? A study on immediate mental health status and psychoneuroimmunity prevention measures of Chinese workforce. Brain Behav Immun.

[45] Adebayo AM, Sekoni OO, Uchendu OC, Ojifinni OO, Akindele AO, Adediran OS (2019). Quality of implementation of the school health program in a rural district of Oyo state, Nigeria: a public-private comparison. J Public Health.

[46] Oyinlade OA, Ogunkunle OO, Olanrewaju DM (2014). An evaluation of school health services in Sagamu, Nigeria. Niger J Clin Pract.

[47] Tran BX, Phan HT, TPT N, Hoang MT, Vu GT, Lei HT (2020). Reaching further by Village Health Collaborators: The informal health taskforce of Vietnam for COVID-19 responses. J Glob Health.

[48] Country Profile: Nigeria community health programs. 2014 [cited 2021 May 27]. Available from: https://www.advancingpartners.org/sites/default/files/landscape/countries/profiles/country_profile_nigeria.pdf

[49] Ordinioha B, Onyenaporo C (2010). Experience with the use of community health extension workers in primary care, in a private rural health care institution in South-South Nigeria. Ann Afr Med.

